# Immunotherapy in the Precision Medicine Era: Melanoma and Beyond

**DOI:** 10.1371/journal.pmed.1002196

**Published:** 2016-12-13

**Authors:** Mack Y. Su, David E. Fisher

**Affiliations:** Cutaneous Biology Research Center, Department of Dermatology, Massachusetts General Hospital, Harvard Medical School, Boston, Massachusetts, United States of America

## Abstract

In a Perspective, Mack Su and David Fisher discuss the development of immunotherapies for treatment of melanoma and other cancer types.

Innovations in cancer immunotherapy over the past decade have reinvigorated the field, which currently stands poised to transform treatment of certain important cancer types. While recent discoveries in immunotherapy have occurred in an era of intense focus on precision medicine—spurred in large part by advances in sequencing technologies allowing for unprecedented insights into individual and tumor genomes—the field of cancer immunotherapy is rooted in a history that is anything but precise. The idea that the immune system could be used to target cancer has existed for over a century. In the 1890s, William Coley provided a spark by noting a connection between spontaneous tumor regressions and concurrent bacterial infections [1]. Suspecting an immune mechanism, Coley and many others in the decades that followed experimented with live bacteria, toxins, vaccines, and countless other agents to coax the immune system to attack cancer, yielding only a tiny number of positive responses and little understanding of the underlying immune mechanisms. As recently as 2010, immunotherapy remained a strikingly imprecise endeavor, with the only United States Food and Drug Administration (FDA)-approved cancer immunotherapies being a pair of systemic cytokines—interferon-α and interleukin-2—and intravesical bacillus Calmette-Guérin (BCG). However, as recent successes reshape the field, efforts to comprehend and harness precise mechanisms in immunotherapy will be required.

## Breakthroughs in Melanoma

**Fig 1:**
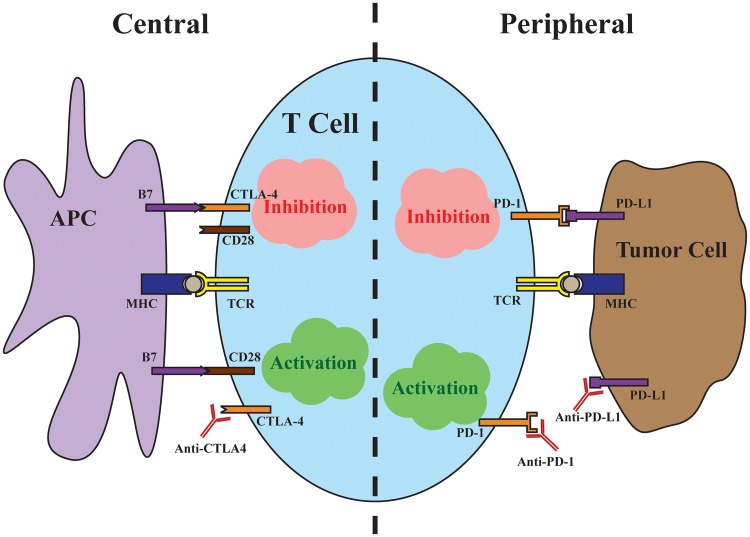
Central and peripheral immune checkpoints. Central immune inhibition occurs as a result of CTLA-4 binding to the costimulatory B7 ligands found on antigen-presenting cells (APCs) with high affinity, preventing signaling through CD28. Antibodies targeting CTLA-4 release this checkpoint to allow T cell activation. Peripheral immune inhibition occurs through binding of the PD-L1 found on tumor cells to the PD-1 receptor on T cells. Antibodies targeting either the ligand or the receptor release this checkpoint.

Perhaps the most profound impacts of both immunotherapy and precision medicine, in the form of targeted therapy, can be appreciated in the transformation of treatment for metastatic melanoma during the past decade. The discovery of the oncogenic V600E driver mutation in BRAF, which is present in ~50% of melanomas, led to the development of vemurafenib and dabrafenib, which are inhibitors of the mutant BRAF kinase [2]. Although targeting mutant BRAF kinase and inhibiting the downstream mitogen-activated protein kinase (MAPK) pathway produces high response rates in melanoma patients, the duration of responses is brief [3], highlighting the need for additional therapies. Meanwhile, preclinical evidence suggested that blocking negative regulators, or checkpoints, of T cell responses could improve antitumor immune responses (Fig 1). The first such negative regulator tested was cytotoxic T-lymphocyte-associated protein 4 (CTLA-4), a T cell surface receptor that binds with high affinity to the costimulatory ligands B7-1 and B7-2 found on the surface of antigen-presenting cells. The interaction with CTLA-4 prevents binding of the costimulatory ligands to cluster of differentiation 28 (CD28), a major costimulatory receptor on the T cell necessary for a robust T cell response. Despite concerns about uncontrollable autoimmunity resulting from systemic CTLA-4 blockade, ipilimumab, a monoclonal antibody against CTLA-4, improved overall survival in patients with metastatic melanoma [4].

The successes of ipilimumab were quickly followed by trials targeting the immune inhibitory interaction between programmed cell death protein 1 (PD-1), found on T cells, and its ligand, PD-L1, found on tumor cells. In contrast to the central immune inhibitory effects of CTLA-4, inhibition through PD-1 predominantly occurs at the periphery, with tumor cells up-regulating PD-L1 in response to local immune signals such as interferon-γ. In a major head-to-head clinical trial, pembrolizumab, a monoclonal antibody against PD-1, demonstrated improved progression-free and overall survival with fewer high-grade adverse events compared to ipilimumab [5]. Importantly, in a minority of cases, checkpoint inhibition produces strikingly durable responses, with up to a third of advanced melanoma patients alive at five years of follow-up [6]. The majority, unfortunately, fail to respond to immunotherapy, with objective response rates to checkpoint inhibitor monotherapy ranging from 10%–40%, with PD-1/PD-L1 targeted antibodies benefiting larger proportions of patients [4,5,7]. Because the mechanisms of CTLA-4 and PD-1 blockade are distinct and potentially complementary, dual checkpoint blockade increases response rates to around 60%, albeit with significantly more adverse events [8,9]. In a matter of a few years, immune checkpoint blockade has become first-line therapy for advanced melanoma and transformed the outlook for patients. Looking ahead, uncovering predictive markers of response to immunotherapy and ultimately understanding the mechanisms of response will be necessary to extend the promise of immunotherapy to broader groups of cancer patients.

Efforts to uncover the precise mechanisms of immune checkpoint inhibition and identify patients likely to benefit from treatment are well under way. Pathologists have long recognized the prognostic significance of tumor-infiltrating lymphocytes [10], and, unsurprisingly, pretreatment CD8+ T cell density is associated with favorable response to immune checkpoint blockade [11]. However, preclinical data suggest that CD8+ T cells in the tumor microenvironment can promote immune inhibition as a result of interferon-γ-mediated induction of PD-L1 on tumor cells [12]. Response to anti-PD1 therapy in melanoma as well as other cancers has been suggested to positively correlate with tumor expression of PD-L1 [13,14], highlighting the central role of this ligand in tumor immune evasion, although PD-L1 expression thus far appears to be an imperfect predictive biomarker of response. A comprehensive understanding of immunotherapy requires a deeper analysis of the interaction between immune system and tumor.

## Genomics-Guided Advances

The rapid progress in cancer genomics in recent years has enabled a close examination of the genetic makeup of thousands of individual cancers across the entire spectrum of major cancer types. Amidst the search for mutations responsible for driving carcinogenesis, it has become evident that overall mutation rates vary dramatically both within and between cancer types [15]. While the vast majority of mutations contribute little, if at all, to intrinsic biological function in tumor development and survival, they represent novel, “non-self” epitopes that, if processed and presented, carry the potential to elicit a tumor-specific immune response [16]. By mining exome sequencing data to predict neoantigens present in a tumor, several groups have demonstrated a strong correlation between total neoantigen burden and positive response to immune checkpoint blockade [17,18]. These insights provide a mechanistic rationale to expand checkpoint inhibition to mismatch repair-deficient cancers of any type, because the dramatically elevated mutation rates in these cancers produce significantly more neoantigens and the potential for a more robust immune response [19].

Although the importance of neoantigens is now firmly established, substantial overlap in neoantigen burden exists between nonresponders and responders, highlighting the need to more precisely identify the tumor-specific antigens required for a successful immune response. Emerging evidence suggests that clonal neoantigens—that is, neoantigens present in all cancer cells—rather than total neoantigen load are critical for T cell recognition and clinical benefit from checkpoint blockade [20]. The critical neoantigens also need not be shared between patients. One targeted approach with the potential to improve and increase responses to checkpoint inhibition is to produce personalized vaccines specific to the neoantigens present in a patient’s tumor [16,21].

However, selecting and manufacturing the optimal neoantigen vaccines within a clinically meaningful time frame remains challenging, and successful targeting of tumor neoantigens requires antigen-specific T cells, which may not be present. Indeed, the efficacy of CTLA-4 blockade likely stems in part from an expansion of the peripheral T cell receptor (TCR) repertoire [22], which presumably includes neoantigen-specific TCRs. Nascent experimental and computational methods may help to resolve the critical TCR–antigen interactions in each tumor [23,24], providing the critical blueprints for delivering precision immunotherapy.

As the precise mechanisms of immune checkpoint inhibition continue to be unraveled, great interest has emerged in combining checkpoint blockade with current targeted therapies. Substantial research on BRAF mutant melanoma indicates that BRAF inhibition synergizes with immune checkpoint blockade by facilitating T cell recognition of melanoma antigens in a more favorable tumor microenvironment [25,26]. Although one early trial combining immune checkpoint blockade with BRAF inhibition stalled because of hepatotoxicity [27], many additional trials are ongoing to optimize dosing and sequencing of the combination. An attractive feature of combining immune checkpoint blockade with targeted therapy is the potential to leverage current advances in precision medicine to impact many cancer types. For example, in BRCA1-deficient ovarian cancer, inhibition of poly(ADP-ribose) polymerases (PARPs), a family of enzymes involved in DNA repair, markedly increases mutational load in tumor cells and has demonstrated synergistic potential with CTLA-4 blockade [28]. Targeting driver mutations such as those in the genes encoding *c-KIT* or epidermal growth factor receptor (*EGFR*) with specific inhibitors appears to foster antitumor immunity [29,30]. In many cancers, disrupting tumor angiogenesis by inhibiting vascular endothelial growth factor (VEGF)/vascular endothelial growth factor receptor (VEGFR) promotes immune cell recruitment and infiltration, which may derive additional benefit from checkpoint inhibition [31]. In all of these cases and others, clinical trials are ongoing to determine the ability of targeted therapies to potentiate response to immune checkpoint inhibition.

## Future of Precision Immunotherapy

Propelled by recent successes, enthusiasm for immunotherapy has surged beyond targeting CTLA-4 and PD-1 pathways. Many additional immune regulators, both stimulatory and inhibitory, are being explored as potential targets for cancer immunotherapy, each with a unique set of advantages and potential risks. Novel insights into neoantigens and combination with immune checkpoint inhibition have breathed new life into cancer vaccines, which had been tested for decades with little success. In addition, cell-based therapies may be required in some patients to elicit a robust antitumor response. Adoptive cell transfer of autologous T cells selected for tumor reactivity [32] and genetically engineered T cells with chimeric antigen receptors targeting tumor antigens [33] have demonstrated promise in melanoma and other cancer types. As new immunotherapies expand the arsenal of cancer therapies, deeper mechanistic insight will be required to inform clinical decisions. The exciting advances toward understanding and delivering precision immunotherapy are poised to change the way cancer is treated.

## Abbreviations

APC: antigen-presenting cell
BCG: bacillus Calmette-Guérin
CD28: cluster of differentiation 28
CTLA-4: cytotoxic T-lymphocyte-associated protein 4
EGFR: epidermal growth factor receptor
FDA: Food and Drug Administration
MAPK: mitogen-activated protein kinase
PARP: poly(ADP-ribose) polymerase
PD-1: programmed cell death protein 1
PD-L1: programmed cell death-ligand 1
TCR: T cell receptor
VEGF: vascular endothelial growth factor
VEGFR: vascular endothelial growth factor receptor
